# Supplementary material for: Genetic Control of GCF Exudation: Innate Immunity Genes and Periodontitis Susceptibility
Source: Int J Mol Sci. 2023 Sep 18;24(18):14249. doi: 10.3390/ijms241814249 (PMC10532312; doi:10.3390/ijms241814249)
Supplement: Supplementary file 1 [file ijms-24-14249-s001.zip › ijms-2431056-supplementary.pdf]

Supplementary Materials Items 1 through 4.

Contents:

S1. **Supporting information from Reference 11**

S1.A. **Figure S1: Protocol for partial human experimental gingivitis study.**

S1.B. **Participant Selection and Examination.**

S1.C. **GCF Measurement.**

S1.D. **Measurement of Cadaverine and Lysine**

S1.E. **Clinical Measurement Variability.**

S1.F. **Statistical Analyses.**

S1.G. **Figure S2. Relationship of plaque index (PI) and gingival crevicular fluid (GCF) to lysine content of dental biofilms after one week of experimental gingivitis.**

S2. **Figure S3. The complete version of Figure 4.**

S3. **Comment. SNP at IL1B+3954 does not change the encoded amino acid.**

S4. **Figure S4. Figure 3 with genetic data from Table 4.**

Supplement

S1. The following supporting information from Reference 11 (Lohinai, Z.; Keremi, B.; Szoko, E.; Tabi, T.; Szabo, C.; Tulassay, Z.; Levine, M. Bacterial lysine decarboxylase influences human dental biofilm lysine content, biofilm accumulation, and subclinical gingival inflammation. J Periodontol 2012, 83, 1048-1056, doi:10.1902/jop.2011.110474) can be downloaded at: www.mdpi.com/xxx/s1.

S1.A. Figure S1: Protocol for partial human experimental gingivitis study.

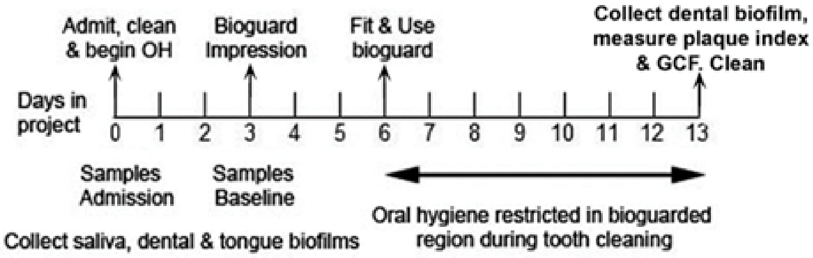

S1.B. Participant Selection and Examination

The study was conducted in Hungary where Dr Lohinai was Principal Investigator. The study was monitored in accordance with the ICH note for guidance on Good Clinical Practice and the Helsinki declaration. The human protocol was reviewed and approved by the Ethics Committee of the Hungarian Medical Research Council (Approval # 11878-1/2006-1017EKL). Prior to participation, the purpose and risks of the investigation were fully

explained to all participants. Subjects were entered into the study only after having given written consent.

Sixteen subjects, 7 men and 9 women were enrolled and completed the project: 12 aged 19 to 24 years and 4 (2 men and 2 women) aged 29, 30, 36 and 38 years. Inclusion requirements were at least 26 teeth, no existing or prior medical conditions and no drug therapy or smoking tobacco. Dental exclusion categories were partially erupted wisdom teeth, cavities requiring treatment, a pocket with a probing depth >2 mm, >10% of teeth with gingival sulci that bled on probing, and the presence of gingival recession or calculus. The protocol is summarized in Figure S1 above.

At admission, saliva was collected into a sterile 10 ml centrifuge tube for 5 min. Biofilm from the upper surface of the tongue or from the gingival surfaces of teeth was collected with a plastic tongue scraper or curette as appropriate (admission samples). After a thorough professional cleaning, an upper right or left quadrant was randomly selected. An impression was taken and a bioguard was fabricated to restrict oral hygiene in this quadrant. The participants were then given a new medium toothbrush and toothpaste, instructed to brush thrice daily at home, and to return after 3 days without cleaning their teeth that morning.

At that visit, saliva, tongue, and dental biofilm samples were again collected (baseline samples), the teeth professionally cleaned, and participants practiced using the bioguard prior to its use when tooth cleaning. They were also told to avoid all additional oral hygiene procedures. At the return visit after 7 days, all subjects were examined by the same experienced clinician (ZL) to reduce inter-examiner variability in clinical measurement. The bioguarded region was isolated from saliva with cotton rolls, gently air-dried, and the buccal and mesiobuccal surfaces of the upper second incisor, first premolar, and first molar scored for plaque index.

S1.C. GCF Measurement

Because a Periotron was not available, GCF exudation was measured by the method of Loe & Holm-Pedersen. Each subject rinsed their mouth with water and the bioguarded region was air-dried and isolated. The examiner then gently inserted a paper strip (2 mm wide) into the gingival sulci at each of the three buccal surfaces from which the plaque index was obtained. The strip was left undisturbed for exactly 3 min to ensure adequate fluid uptake and the wetted length immediately measured with a caliper to avoid fluid loss. The wetted length of the paper strip was multiplied by its width and liquid load (0.22 μg/mm2) and divided by 3 to obtain the GCF exudation volume in μL/min. Dental biofilm was then collected from the bioguarded region and the teeth were professionally cleaned.

S1.D. Measurement of Cadaverine and Lysine

Cadaverine and lysine were determined as described previously by our group. Biofilm samples were placed in pre-weighed micro-centrifuge tubes and weighed using an analytical balance. Biofilm and saliva samples were diluted with 10 times their wet weight of 65 mM NaCl. The saliva samples were centrifuged (10,000 g, 10 min, 4°C), but the biofilm samples were first homogenized in the micro-centrifuge tubes using a pestle. A homolog of cadaverine and putrescine (1,3-diaminopropane) was added to each supernatant to 25 μM (internal standard), followed by a double volume of acetonitrile to precipitate protein. After centrifugation, the supernatants were made to 50 mM borate buffer pH 8.0. Ethanolic 4-fluoro-7-nitrobenzofurazan (NBD-F) was added to 0.05 mg/mL and incubated at 65°C for 15 min. An equal volume of water was added, and the solutions were stored at 4°C. Samples were introduced into 20 cm fused silica capillaries under pressure (5 sec, 0.5 psi, 15°C)

and separated at 70 µA in 0.03 M phosphate buffer, pH 2.2. NBD-derivatives were separated using a capillary electrophoresis system controlled by 32 Karat software version 5.0 coupled to an argon-ion, laser-induced fluorescence detector and identified by emission at 520 nm after excitation at 488 nm. The amine derivatives (0 – 50 µmol) were used to construct linear calibration curves. Losses were adjusted by internal standard recovery. Assay accuracy and precision were 6% as reported in detail previously [26]. In addition, because lysine is the sole source of cadaverine which is stable in the oral cavity, totaling the cadaverine plus lysine content of dental biofilm indicates lysine converted to cadaverine plus lysine remaining ('total' lysine content). The cadaverine fraction (CF) of this 'total' indicates lysine decarboxylase activity.

S1.E. Clinical Measurement Variability.

For each participant, plaque index was the mean from six bioguarded teeth surfaces and GCF the mean from three such surfaces. The standard deviation about each mean indicated the variability of the respective measurements within each participant. Because these standard deviations were not normally distributed, an estimate of measurement variability was obtained by converting each standard deviation to a percentage of its respective mean and identifying the median, 35.9% for plaque index and 36.5% for GCF.

S1.F. Statistical Analyses.

Differences in mean cadaverine and lysine contents and in cadaverine fraction of dental biofilm, saliva and tongue biofilm between admission and baseline and, independently, in dental biofilm from baseline to the end of oral hygiene restriction, were compared using non-parametric tests. Relationships between lysine and cadaverine within dental biofilm, saliva and tongue biofilm at baseline, and of lysine and cadaverine in dental biofilm to plaque index and GCF after oral hygiene restriction were explored. Spearman's ranked coefficients of determination squared (r2) and significance (p) were obtained initially. Significant relationships ($p < 0.05$) were explored further using least squares multiple and non-linear regressions as appropriate. Standardized residuals were checked for normality to ensure that assumptions were not violated by the non-normal distribution of measurements in the population. Spearman correlations had a subjects-to-independent variable ratio that was 16:1, close to optimum for a search for unknown relationships. (The Princeton University Data and Statistical Services: http://dss.wikidot.com/assumptions-of-regression). Parametric least squares regressions used no more than 2 variables and had a subjects-to-independent variable ratio that was greater than the accepted minimum of 5:1. Squared correlation coefficients (R2) were adjusted for number of variables and data points in the model.

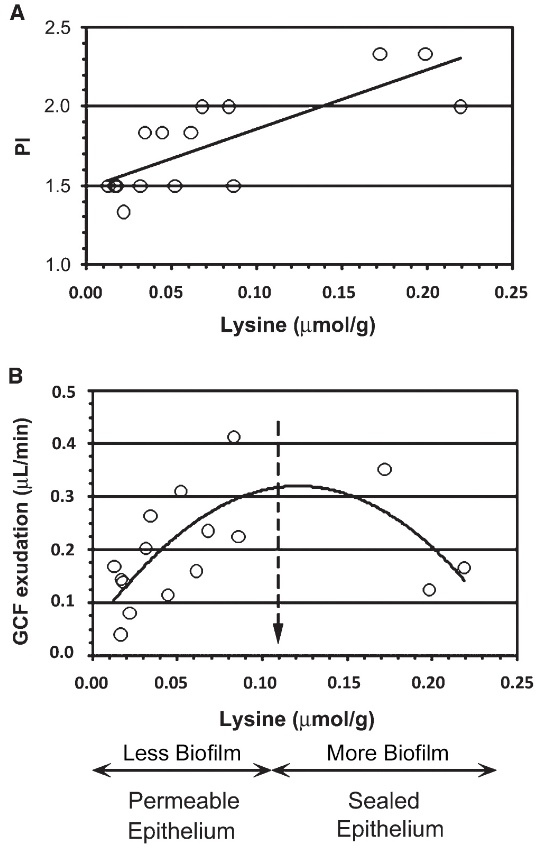

S1.G. Figure S2. Relationship of plaque index (PI) and gingival crevicular fluid (GCF) to lysine content of dental biofilms after one week of experimental gingivitis.

A) PI correlated with biofilm lysine (Lys) content after oral hygiene restriction. Spearman $R^2 = 0.58$, $P < 0.001$, the result of a least-squares regression whose equation was as follows: PI = 1.48 + 3.75 * Lys; $R^2 = 0.61$, df = 1,14, ANOVA, F statistic= 24.1, $P < 0.001$ (Section S1F). If the three high lysine values (values exceeding 0.11 mg/mL) are removed, the association remains significant (Spearman $R^2 = 0.33$, P =0.042).

B) GCF exudation rate regressed on biofilm lysine content after oral hygiene restriction. Vertical arrow indicates the minimal lysine concentration of normal blood plasma (0.11mg/mL [11]. A quadratic relationship was significant: least-squares regression $R^2 = 0.37$, df = 2,13, ANOVA, F = 5.4, $P < 0.02$. GCF = $-18.5*\text{Lys}^2 + 4.5*\text{Lys}+0.05$. Coefficients: Lys^2 ‘t’ statistic = -3.09, $P < 0.01$; Lys ‘t’ statistic = 3.27, $P < 0.01$; constant t statistic = 1.01, not significant.

S2. Figure S3: The complete version of Figure 4.

A	ia	ib	ic	id	ii	iii	iv	v	vi	vii	viii	ix	x	xi	
Host	Sex	Lys	GCF	PII-1	S/W	*IL1B* -511	*IL1B* +3954	*IL10* -1082	*IL6* -1363	*IL10* -597	*CD14* -260	*IL1A* -889	*COX2* +8473	*MMP8* -799	Comments
1	M	0.013	0.168	1.500	S	G/A	C/T[ed]	T/T	G/T[ed]	G/T	A/G	G/G	A/A	A/A[e]	Epistasis[e] dominant[d]
2	F	0.017	0.145	1.500	S	G/A	C/T[s]	C/C[s]	G/G	G/G	A/G	G/G	G/G	G/A	Specifies[s]
3	M	0.052	0.310	1.500	S	G/A	C/T[s]	C/C[s]	G/G	G/G	G/G	G/G	A/G[s]	G/A	[*COX2* +8473(AG)][s*]
4	F	0.031	0.202	1.500	S	G/A	C/C	C/C	G/G	G/G	G/G	G/G	A/G	G/G	
5	F	0.172	0.351	2.333	S	G/A	C/C	T/C	G/G	G/T	A/G	G/G	A/A	G/G	
6	F	0.018	0.139	1.500	S	G/A	C/C	T/T	G/G	G/T	A/G	G/A	A/A	G/A	
7	F	0.034	0.264	1.833	S	G/A	C/C	C/C	G/G	G/G	G/G	A/A	A/G	G/A	
8	F	0.083	0.412	2.000	S	A/A	C/C	C/C	G/G	G/G	G/G	G/A	A/A	G/A	
9	M	0.219	0.165	2.000	S	A/A	C/C	C/C	G/G	G/G	G/G	G/A	A/A	G/A	
10	M	0.086	0.224	1.500	W	G/A	C/T[s]	T/T[s]	G/G	T/T[s]	G/G	G/G	A/A	G/A	[*IL10* -597(TT)][s*]
11	M	0.017	0.041	1.500	W	G/A	C/C[eg]	T/T	G/T[eg]	G/T	G/G	G/A[eg]	G/G	G/G	[e]Tooth Loss (TL)[g]
12	F	0.044	0.115	1.833	W	G/A	C/T[s]	C/C	G/G	G/G	A/A[s]	G/G	A/A	G/G	Specifies[s]
13	M	0.022	0.081	1.333	W	G/G	C/T	T/C	G/G	G/G	A/G	G/G	G/G	G/G	
14	M	0.061	0.159	1.833	W	G/G	C/T[g]	T/C	G/G	G/G	A/G	G/A[g]	G/G	G/A	TL[g]
15	F	0.199	0.124	2.333	W	G/G	T/T	T/C	G/G	G/T	G/G	G/G	A/A	G/A	
16	F	0.068	0.235	2.000	W	No DNA									

S3. Figure S4: Figure 3 with genetic data from Table 4.

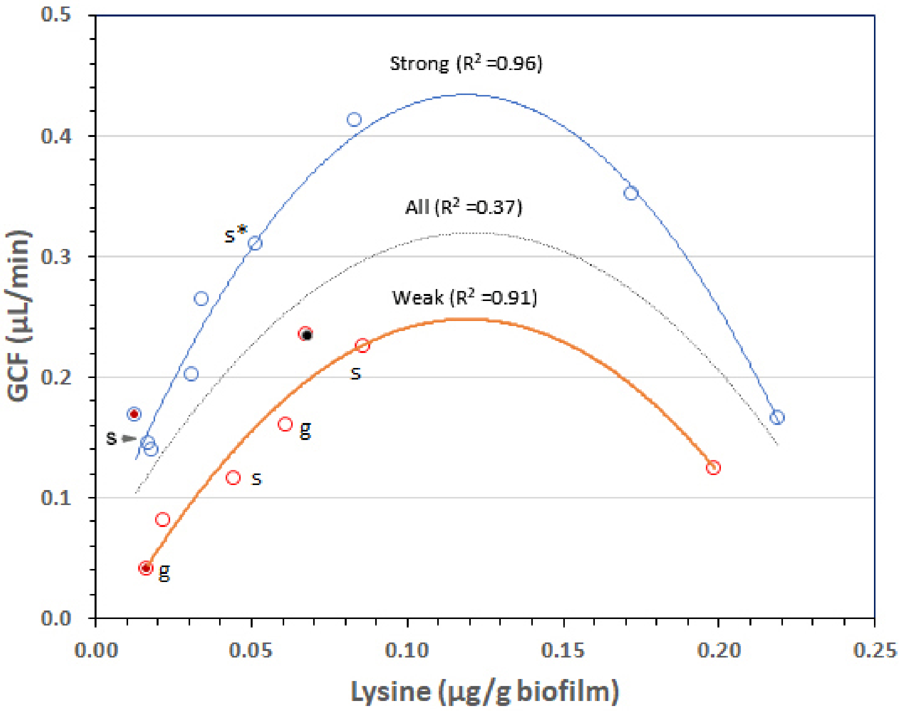

Circles not filled and without the letter 's' indicate 6 strong and 3 weak hosts determined from alleles of *IL1B*+3954 and *IL1B*-511 alone. The circle filled with a black dot indicates an EG host who was abroad and unavailable for DNA collection. The 2 circles, filled with a red dot on the far left indicate two epistatic responders (Section 2.2). The 4 circles indicated by the letter 's' indicate the need for the allele of a second gene to specify whether the GCF is strong or weak, and the asterisk (*) indicates the presence of an alternative specifying allele from a different second gene (Section 2.1). Annotation 'g'

refers to the tooth loss genotype [8]. This figure was presented in a poster at the EuroPerio10 conference in Copenhagen in July 2022.

S4. Location of SNP at IL1B+3954 (rs1143634) indicates no difference in the encoded amino acid.

The two genes of IL1 are encoded on Chromosome 2, and the SNP at IL1B+3954 is present on exon 5 of the gene. The reference SNP at this site is cytidine (C) and the alternate SNP is thymidine (T). The upstream portion of the codon containing the SNP is TT. In the reference DNA, TTC is transcribed to UUC in the RNA transcript, and the alternate sequence to UUU. These codons are both translated as phenylanaline (F or Phe). This means that the same amino acid is transcribed from both the reference and alternate codons containing the C or T SNP at IL1B+3954. The two SNPs must therefore have another function, which may be the epstatic interaction with IL6-1363 (see Discussion, section 3.1).

Source: Go to http://www.ensembl.org/id/ENSG00000125538 On the far left near the foot of the page under 'Genetic variation' press 'variant table.' Then press 'edit,' select 'find in page' and put in 'rs114' to take you to the information about what the SNP at this site encodes (pathway accessed 30/8/2023).
